# Tissue characteristics in a traumatised immature necrotic incisor after unsuccessful regenerative endodontic treatment: an immunohistological case report

**DOI:** 10.1007/s40368-026-01190-2

**Published:** 2026-03-09

**Authors:** A. Wikström, N. Tewari, M. Anderson, M. Brundin, G. Tsilingaridis

**Affiliations:** 1 Division of Peadiatric Dentistry, Department of Dental Medicine, Karolinska Institute, Huddinge, Sweden; 2 Department of Endodontics, Eastman Institute, Public Dental Services, Stockholm, Sweden; 3 Centre of Paediatric Oral Health Research, Stockholm, Sweden; 4 Division of Pediatric and Preventive Dentistry, Centre for Dental Education and Research, All India Institute of Medical Sciences, New Delhi, India; 5 Department of Paediatric Dentistry, Eastman Institute, Public Dental Services, Stockholm, Sweden; 6 Department of Endodontics, Umeå University, Umeå, Sweden

**Keywords:** Regenerative endodontic treatment, Immunohistological analysis, Traumatised immature necrotic tooth, Pulp necrosis, Regenerated odontogenic tissues, Cholesterol crystals

## Abstract

**Purpose:**

The present case report aimed to present an immunohistological analysis of newly formed intracanal tissues in a traumatised immature permanent incisor with pulp necrosis and apical periodontitis following RET.

**Case description:**

A 7-year-old girl sustained a combined traumatic dental injury, an uncomplicated crown fracture and lateral luxation, affecting an immature maxillary central incisor. The tooth subsequently developed pulpal necrosis and apical periodontitis and was treated with RET using calcium hydroxide as an intracanal medicament. A blood clot served as the scaffold, followed by placement of calcium silicate cement (Biodentine^®^) and a definitive resin composite restoration. During follow-up, the tooth developed ankylosis with progressive infraocclusion. At 13 years of age, the tooth was extracted, and prosthetic rehabilitation was completed using a resin-bonded Rochette bridge.

**Results:**

Histological and immunohistological analyses revealed a heterogeneous mixture of mineralised and connective tissues within the root canal space. The tissues demonstrated infiltration of fibroblast-like cells, resorptive cells, blood vessels and nerve fibres. Numerous fibrous cystic structures containing cholesterol crystals were identified in the middle third of the root canal.

**Conclusion:**

The newly formed intracanal tissues demonstrated limited similarity to intact odontogenic tissues. Both inflammatory root resorption and ankylosis-related replacement resorption, likely associated with the initial luxation injury, were observed. The presence of cholesterol crystal deposits represents a novel finding that may provide new insights into the biological processes occurring after RET. As cellular events following RET cannot be fully controlled clinically, the risk of unfavourable outcomes should be considered carefully, particularly in cases involving luxation injuries.

## Introduction

Traumatic dental injuries (TDIs) constitute a significant global health problem, affecting approximately one billion people worldwide (Petti et al. 2018); TDIs to the permanent dentition most frequently occur at a young age, when tooth development is still incomplete. As long as the dental pulp remains vital, the tooth maintains a functional defence barrier against bacterial invasion. In immature teeth with a wide-open apical foramen, the pulp exhibits a greater capacity to survive trauma and its sequelae (Robertson et al. 2000). Reestablishment of vascular and neural supply may allow continued root maturation without the need for endodontic intervention. Nevertheless, immature teeth remain at risk of post-traumatic pulpal breakdown and infection. Bacterial species colonising necrotic pulp tissue are known to induce periapical inflammation and apical periodontitis (Möller et al. 1981). In addition, traumatic injuries to the periodontal ligament and to the incompletely mineralised layers of pre-dentine and pre-cementum lining the internal and external tooth surfaces may result in other complications, such as root resorption (Abbott 2016). The most common post-traumatic resorptive processes include external inflammatory root resorption and replacement resorption, also known as ankylosis (Andreasen et al. 1995).

The endodontic management of traumatised immature permanent teeth with pulp necrosis and apical periodontitis presents a particular clinical challenge. The presence of an open apex, a wide root canal and thin dentinal walls necessitates specialised treatment approaches. These challenges include achieving adequate disinfection of the root canal system, preventing extrusion of endodontic medicaments into the surrounding tissues, and ensuring optimal placement of obturating materials in the wide apical region, with an inherent risk of overfilling.

Current treatment options for necrotic immature teeth include apexification and regenerative endodontic treatment (RET). Conventional apexification techniques involve the use of calcium hydroxide or mineral trioxide aggregate (MTA). Studies have reported comparable success rates between these techniques with respect to periapical healing and apical hard tissue barrier formation (Lin et al. 2016). However, neither approach promotes continued root maturation, leaving the affected tooth structurally fragile and more susceptible to fracture. Consequently, RET has been introduced as an alternative treatment modality aimed at strengthening the tooth structure through stimulation of continued root development (Nagata et al. 2014). The ideal goal of RET is to “replace damaged structures, including dentin and root structures, as well as cells of the pulp–dentin complex” (Murray et al. 2007).

From a clinical perspective, successful RET outcomes include an asymptomatic tooth with no clinical or radiographic signs of pathology, such as fistula, abscess, tenderness to percussion or palpation or periapical radiolucency, combined with evidence of continued root maturation, including increases in root length, dentinal wall thickness and apical closure. However, clinical outcomes may vary considerably, ranging from asymptomatic teeth without measurable changes in root dimensions to cases demonstrating varying degrees of continued root maturation (Chen et al. 2012). Furthermore, several studies have indicated that the success of RET in immature teeth with pulp necrosis remains unpredictable (Schmalz et al. 2020).

The relationship between clinical and radiographic outcomes and the histological status of the intracanal tissues following RET is not yet fully understood. Ongoing debate exists regarding the biological nature of the processes occurring after treatment. It has been suggested that clinically applied RET protocols result in the formation of newly deposited intracanal tissues that merely resemble native pulp tissue, indicating that the process represents “repair” rather than true “regeneration” (Lin and Rosenberg 2011).

However, histological data from human teeth treated with RET are only available in situations where the teeth are considered unsalvageable and are analysed after extraction. Therefore, histological evidence remains scarce. There are recent reports on the absence of vital tissue inside the root canal showing radiographic healing of periapical lesions but no or poor root maturation after RET (Nosrat et al. 2013). Consequently, there is a substantial need to expand the current knowledge base regarding the histological characteristics of tissues formed following RET in humans (Iwaya et al. 2001).

Thus, this article reports the management, clinical, radiographic and immunohistological outcomes of RET in a child with traumatised immature maxillary central incisor with pulp necrosis and apical periodontitis.

## Case report

The present case report has been presented in accordance with the PRICE 2020 guidelines for reporting endodontic case reports (Nagendrababu et al. 2020). Prior to treatment, both verbal and written information regarding the procedure was provided, and parental consent was obtained for data use and analysis.

A healthy 13-year-old female presented with a history of traumatic dental injury to the permanent maxillary right central incisor (tooth 11) at age 7 years, caused by a fall after stumbling on a stone. According to the referral documentation, the injury was initially classified as an uncomplicated crown fracture in combination with lateral luxation. At the time of examination in the emergency department, however, the tooth was in normal position with physiological mobility, suggesting that any previous displacement had spontaneously resolved prior to assessment. No splinting was, therefore, indicated. The patient record contained no additional documentation clarifying the clinical findings underlying the initial diagnosis of lateral luxation.

At the time of injury, the tooth responded positively to pulp sensibility testing, and radiographs revealed no pathological findings. The tooth was restored with light-cured resin composite. A follow-up was scheduled 4 months later.

**Fig. 1:**
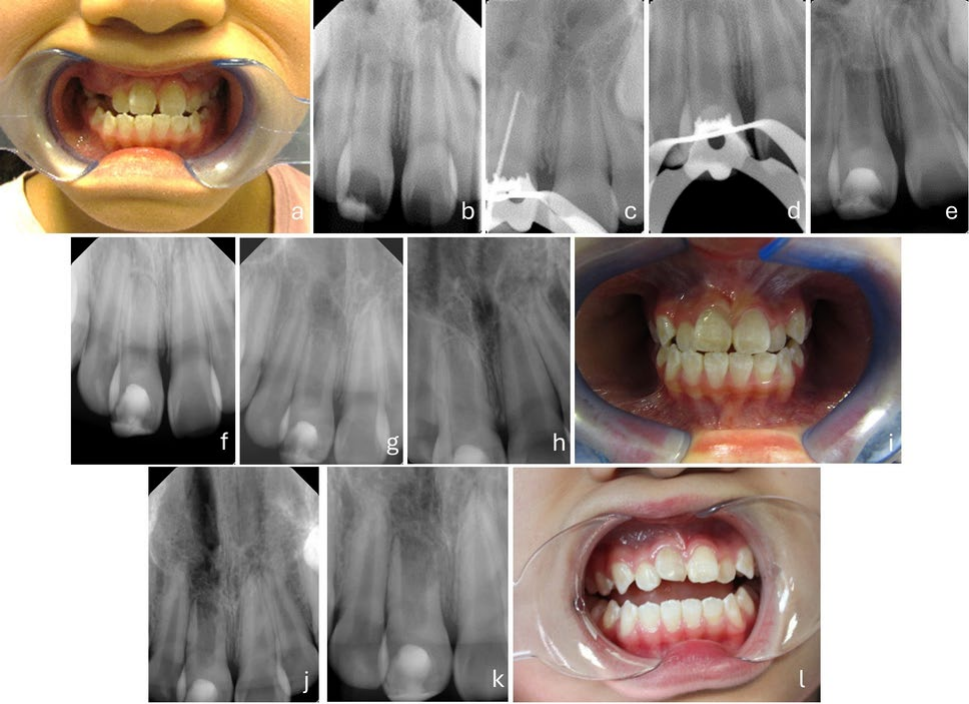
Pre-operative clinical and radiographical findings of tooth 11: **a** clinical and **b** radiographical appearance at the time for examination at the Department of paediatric dentistry at Eastman Institute, Public Dental Health Services, Stockholm, Sweden. Intraoperative and immediate postoperative radiographs of tooth 11: **c** establishment of the working length; **d** placement of the resorbable collagen scaffold and bioceramic material; **e** immediate postoperative radiograph and final restoration; **f**–**i** 2-year follow-up of tooth 11; **j**–**l** 3-year follow-up of tooth 11

At follow-up, the patient presented with buccal gingival swelling, light grey discoloration and increased mobility of tooth 11. The tooth was non-responsive to cold and electric pulp tests (Endo Ice or Endo Frost, Coltene Whaledent Roeko; Langenau, Germany and Pulp Tester Analytic Technology, Richmond, VA, USA), whereas the contralateral tooth 21 responded normally. Radiographic examination revealed incomplete root formation with a widened periodontal ligament space. Based on these findings, tooth 11 was diagnosed with pulp necrosis and apical periodontitis. In addition, tooth 11 was in crossbite, and adjacent teeth were crowded (Fig. 1a, b). Abscess drainage was performed, and the patient was referred to the Department of Paediatric Dentistry at Public Dental Services, Eastmaninstitutet, Folktandvården Stockholm, Sweden,

After multidisciplinary planning, RET was chosen to promote continued root development. The treatment was performed over two appointments as previously described (Wikström et al. 2022). At the first appointment, local infiltration anaesthesia was administered (1.8 mL Citanest Dental Octapressin 3% + 0.54% mg/mL prilocaine hydrochloride + felypressin, Dentsply DeTrey GmbH, Konstanz, Germany). Following rubber dam isolation and disinfection, access was prepared. Necrotic pulp tissue was removed, and the canal was irrigated with 20 mL 0.5% buffered sodium hypochlorite (Dakin’s solution, APL AB, Kungens kurva, Sweden) for 5 min, 2 mm short of the working length (Fig. 1c). Passive ultrasonic agitation was applied without mechanical instrumentation (IRRI S, IRRI K, Endomark Dental AB, Gothenburg, Sweden). The canal was further irrigated with 5 mL 15.3% EDTA (APL AB, Kungens kurva, Sweden) for 5 min, followed by saline irrigation. Calcium hydroxide dressing (Calasept^®^, Directa AB, Upplands Väsby, Sweden) was placed, and the access cavity was restored temporarily with zinc oxide–eugenol cement (Labservice AB, Sundsvall, Sweden) covered by glass ionomer cement (GC Fuji TM IGP Fast, GC Corporation, Tokyo, Japan),

At the second appointment, approximately 4 weeks later, the tooth was asymptomatic. Local anaesthesia without vasoconstrictor was administered (1.8 mL Carbocain Dental, 30 mg/mL, Dentsply Pharmaceuticals DeTrey GmbH, Konstanz, Germany). After isolation and disinfection, the dressing was removed using 5 mL 0.5% sodium hypochlorite, followed by 15 mL EDTA (APL AB, Kungens kurva, Sweden) for 5 min and final irrigation with 2 mL saline (APL AB, Kungens kurva, Sweden). Bleeding was induced from the periapical tissues using a pre-curved hand file until the canal was filled with blood to the cementoenamel junction. After confirming clot stability, a resorbable collagen scaffold (PARASORB^®^ Cone, Resorba Medical GmbH, Nuremberg, Germany) was placed, followed by approximately 2 mm of Biodentine (Septodont^®^, Saint-Maur-des-Fossés, France), Fig. 1d. The access cavity was restored with zinc oxide–eugenol cement, glass ionomer cement, and light-cured resin composite, Fig. 1e.

**Fig. 2:**
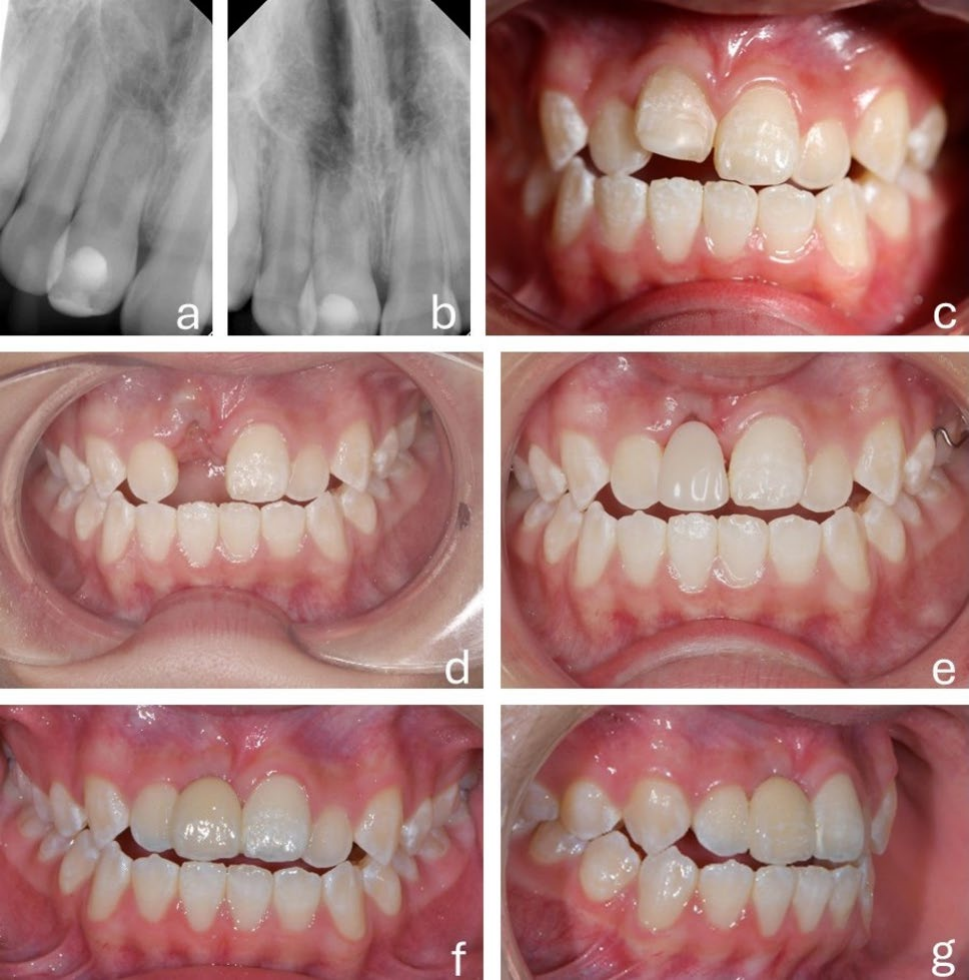
Clinical and radiographical appearance at the final follow-up and after prosthetic treatment: **a**–**c** tooth 11 at 4-year follow-up displaying infra-position and a metallic sound on percussion, suggesting ankylosis. The tooth exhibited further infra-position and progressive root resorption without signs of continued maturation; **d**, **e** clinical appearance after extraction of tooth 11; **f**, **g** postoperative appearance after treatment with resin-bonded Rochette bridge

Follow-up examinations were conducted at 6-month intervals for 4 years. At 6 months and 1 year, no radiographic evidence of continued root formation or apical closure was observed. The tooth remained asymptomatic, and cold and electric pulp tests were negative. Radiographs at 2 years showed no significant root maturation or hard tissue deposition along the root walls, and sensibility tests remained negative, Fig. 1f–j. Similar findings were noted at the 3-year follow-up, with the additional observation of progressive resorption of the mesial root wall, Fig. 1j–l. The tooth displayed infra-position and a metallic sound on percussion, suggesting ankylosis. At 4 years, the tooth exhibited further infra-position and progressive root resorption without signs of continued maturation (Fig. 2a–c).

Given the tooth’s ankylosed and infraoccluded status, intervention was undertaken to maintain the developmental capacity of the alveolus and to prevent bone resorption and space loss that could compromise the eruption of the adjacent teeth. Multidisciplinary planning considered patient compliance, orthodontic factors, and local interventions. Auto-transplantation was not feasible due to complete root development of potential donor premolars. Decoronation was considered; however, uncertainty regarding the nature of the intracanal tissue and apical bone ingrowth made the outcome unpredictable. Extraction and prosthetic rehabilitation with a resin-bonded Rochette bridge were, therefore, planned, preceded by a removable appliance with a temporary tooth for short-term space maintenance. Orthodontic intervention was deferred until age 17 years.

After reviewing the treatment plan and discussing how the procedure would be carried out, the treatment was performed with good acceptance from the patient. Under nitrous oxide sedation and local anaesthesia, tooth 11 was extracted gently and immediately fixed in 10% formaldehyde, Fig. 2d, e. Parental consent was obtained for use of the tooth in research. Prosthetic rehabilitation was completed at a subsequent visit, Fig. 2f, g.

For histological analysis, the crown of tooth 11 containing restorative materials was removed to improve paraffin infiltration. The root was decalcified in 50% formic acid (Saveen & Werner AB, Limhamn, Sweden) and 5 M sodium citrate (pH 6.5, Thermo Fisher Scientific, Delphi, India) for 2 weeks, embedded in paraffin, and longitudinally sectioned to the apical foramen. Semi-serial sections (~ 6 µm thick, 50 – 70 µm intervals) were stained with haematoxylin–eosin (HE) and examined under light microscopy (Olympus BX41, Tokyo, Japan) (Thibodeau et al. 2007).

### Immunohistochemical tissue analysis

Histological analysis was performed independently by two pathologists. Regenerated tissues were evaluated in the following anatomical regions: root dentinal walls, pulp space and apical area. The sections were stained with HE and immunohistochemically with antibodies against CD68, Ladewig, and S-100 protein.

Modified Ladewig trichrome staining was used to visualise overall tissue architecture and to differentiate mineralised dental tissues from soft connective tissues, which is essential for interpreting trauma-related structural changes in the tooth (Nanci 2024; Kim et al. 2018).

Immunohistochemical staining with antibodies against CD68 was performed to identify macrophages and odontoclast/osteoclast-like cells, which play a central role in inflammation and resorptive processes following dental trauma (Pereira et al. 2015).

S-100 protein was selected as a well-established marker for neural and Schwann cell components, allowing assessment of pulpal innervation and neural response to injury (Byers and Närhi 1999). The combined use of histochemical and immunohistochemical methods, therefore, provides complementary structural and cellular information relevant to the biological response of dental tissues after trauma.

At 100 × magnification, original root dentine exhibited regularly organised dentinal tubules, whereas newly formed dentin was irregular and amorphous, with variable tubule width, layering and density. The root canal contained both mineralised and connective tissues, forming resorption canals in the apical half with bone-like cells. No hard tissue ingrowth was observed coronally, where only fibrous tissue was present. The periodontal ligament was largely intact, though areas of resorption were noted.

**Fig. 3:**
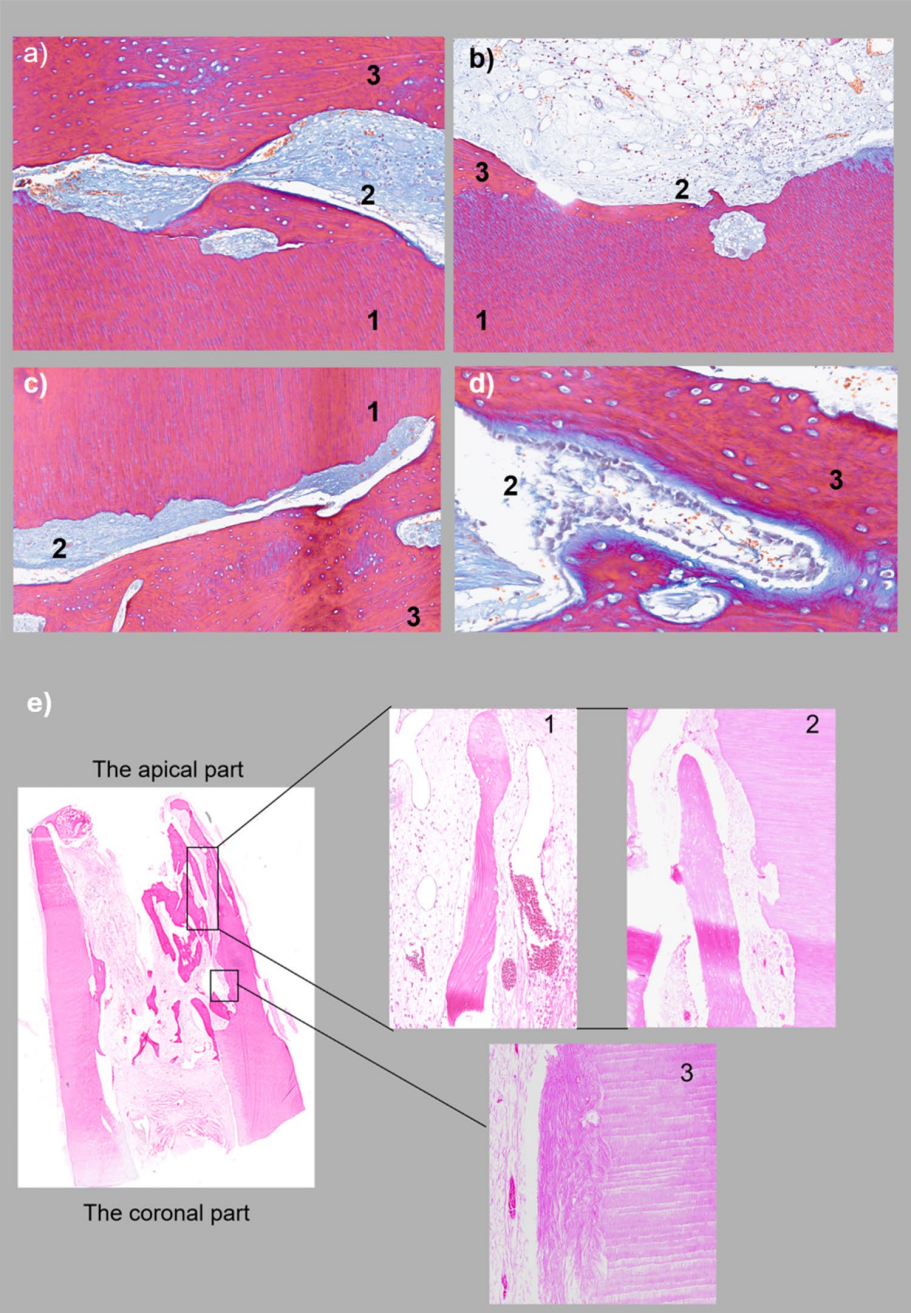
Haematoxylin and eosin staining of the dentinal walls: a micro-section of the root showing the presence of internal resorptive tissue in the apical area. Pictures **a**, **b**, and **c** × 40 magnification showing the 1—tubular structure of the native dentin characterised by regular pattern of the dentin tubules; 2—internal resorptive process; and 3—newly formed dentine with amorphous and irregular tubular structure with few dentin tubules. Picture **d** A higher magnification (× 100) showing the resorption lacunae in the newly formed dentin. Picture **e** Histological images from the LADEWIK staining of the root walls: a micro-section of the root showing the presence of resorptive tissue in the apical area. 1—outlined area showing ingrowth of a mixture of soft and mineralised tissue on the inner dentinal wall; 2—earlier stage of inflammatory root resorption with osteoclasts on the intracanal dentin surface attaching to the dentine forming resorption lacunae; 3—higher (× 100) magnification showing disruption of the odontoblast lining and lining and replacement resorption with ingrowth of bone from the external into the internal root surface

Ladewig staining revealed active resorption on both internal and external root surfaces. External surface resorption presented as lacunae, whilst replacement resorption showed ingrowth of bone cells through the apical foramen. In the apical and mid-root regions, inflammatory root resorption was identified, with osteoclasts present, Fig. 3. Intracanal resorption was observed along the dentinal walls lacking an odontoblastic layer, with more advanced resorption on the mesial than distal aspect.

**Fig. 4:**
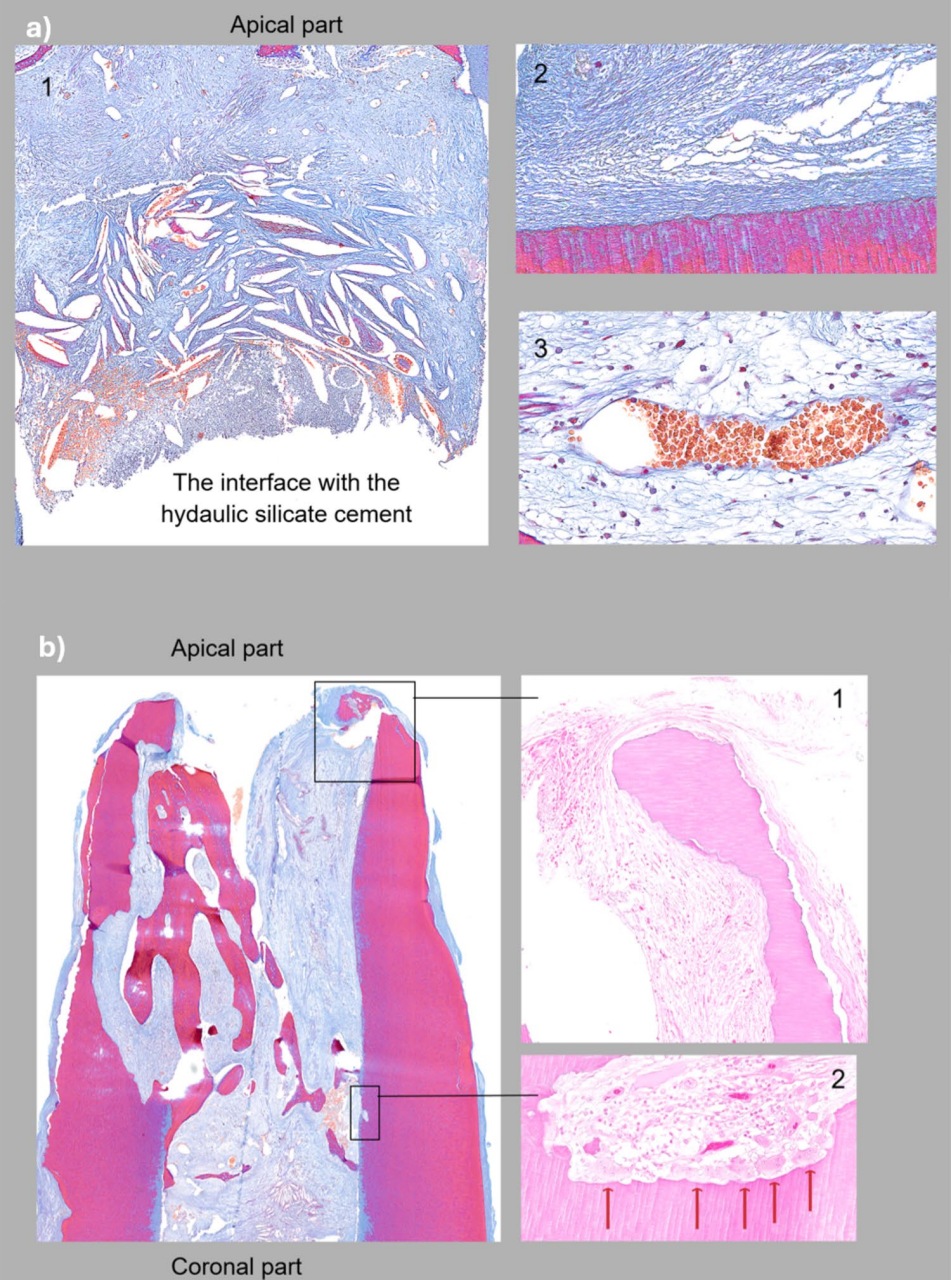
Histological images from the haematoxylin and eosin staining of the pulp space: Part **a** A micro-section of the pulp space showing the presence of fibrous tissue at the interface with dentin. 1—fibrous tissue with numerous fibrous cysts filled with cholesterol crystals; 2—the interface of the connective tissue and dentin and 3—higher (× 100) magnification of the outlined areas showing the vascularization of the connective tissue. Part **b** Histological images from the apical region of the root. A micro-section of the HE staining of the apical region. 1—higher (× 100) magnification of the outlined area showing modest deposition of resorptive tissue at the mesial part of the root tip; 2—higher (× 200) magnification of the outlined area showing ongoing multinucleated osteoclasts resorbing the dentinal wall (red arrows)

Intracanal tissues stained with HE demonstrated abundant vascularisation with extravasated erythrocytes in the deeper pulp layers, Fig. 4a. Fibrous tissue containing numerous fibrous cysts with cholesterol crystal-filled lumens was present, along with islands of mineralised bone-like and dentin-like tissue. LADEWIG staining of the apical region revealed limited deposition of mineralised dentine-like tissue, Fig. 4b, which did not result in apex closure or thickening/elongation of the dentinal walls. External root surface cementum resorption was also observed.

**Fig. 5:**
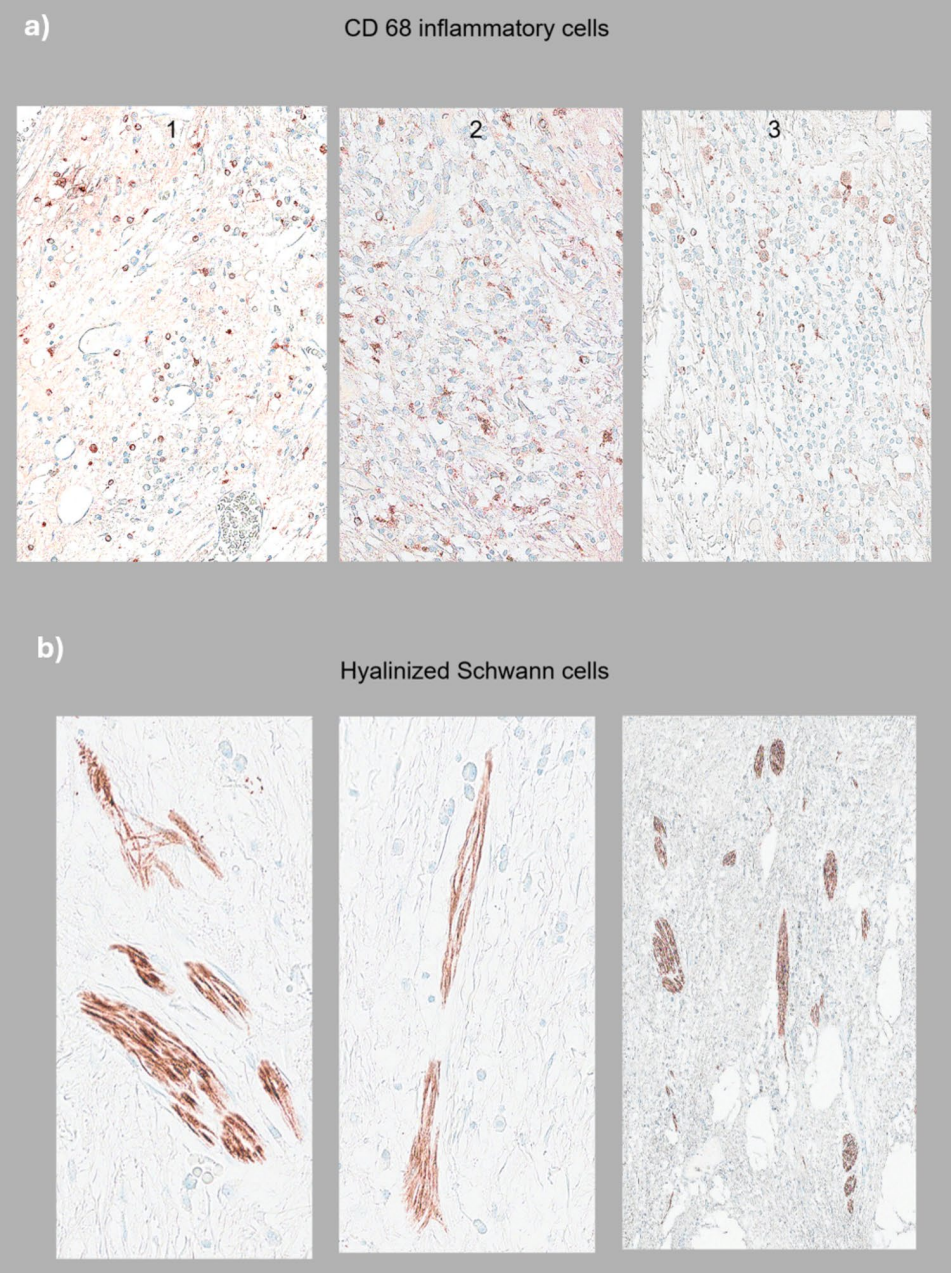
Histological images from the immunohistological staining: Part **a** CD 68 staining of inflammatory cells in the pulp space: A micro-section of different parts of the pulp space, (× 200 magnification) showing conglomerates of inflammatory cells (lymphocytes, macrophages, connective tissue fibroblasts, multinucleated giant cells, and neutrophils). 1—mild inflammation; 2—moderate inflammation; 3—heavy inflammation. Part **b** Histological images from the S-100 staining of the pulp space tissue. 1–2 and 3—A higher (× 200) magnification of the outlined areas showing hyalinised Schwann cells (stained in S-100, brown)

CD68 immunostaining confirmed the presence of inflammatory cells throughout the root canal space, Fig. 5a, including lymphocytes, macrophages, fibroblasts, multinucleated giant cells and neutrophils, indicative of a low-grade inflammatory process; S-100 staining demonstrated hyalinised areas in the deeper pulp regions, suggesting ongoing neurogenesis (Fig. 5b).

## Discussion

### Characterisation and spatial location of the newly formed tissues after RET

In the present case, a mixture of different tissue types was identified, confirming the occurrence of angiogenesis, neurogenesis, replacement root resorption and inflammatory root resorption (Torabinejad et al. 2014; Digka et al. 2020). The newly formed mineralised tissues consisted primarily of bone- and dentine-like tissue deposited along the root walls, predominantly in the apical third of the pulp space. Nevertheless, the newly formed tissues only partially resembled intact odontogenic tissues. This finding is consistent with previous studies reporting apical ingrowth of bone through the apical foramen (Tsilingaridis et al. 2012), supporting the concept that RET often results in repair or a combination of repair and regeneration rather than true tissue regeneration.

Notably, numerous fibrous cysts filled with cholesterol crystals were observed in the middle third of the pulp space. To the authors’ knowledge, this finding has not been reported previously in association with RET. Besides in plaques in cardio-atherosclerotic disease, cholesterol crystals have been demonstrated in remote body sites with pathological conditions, such as systemic bacterial infections, joints affected by gout, cornea or retina in diabetic retinopathy (Abela et al. 2023). Physiologically, cholesterol crystals serve an essential role in the membrane structure or plasma cells and erythrocytes. They influence phospholipid bilayer width, lipid dynamics, raft formation and protein function (Paukner et al. 2022). Nevertheless, within dental research, cholesterol crystals have so far only been reported in periodontal tissues (Nair et al. 1998). The presence of such crystals has been associated with failures due to non-resolving apical periodontitis lesions (Nair 1999).

Currently, it is not possible to explain how and why deposits of cholesterol crystals occurred in the pulp space of the tooth treated with RET. As the crystals may originate from disintegrating erythrocytes and plasma cells within stagnating blood vessels, which, in the present case, may be interpreted as a late failure of the newly established vasculature. The needle-shaped structure of cholesterol crystals may cause direct damage to tissues by disrupting their structure and organisation. In addition, the cholesterol crystals tend to aggregate forming stacks of crystals with similar orientation.

The impact of cholesterol crystals on the outcome of RET is unclear. However, it has been demonstrated that cholesterol crystals are not inert and cause local tissue inflammation. Their pro-inflammatory potential results in monocyte and macrophage infiltration surrounding their edges. Upon immune cell activation, pro-inflammatory cytokines like tumour necrosis factor (TNF) and IL-1β are secreted (Samstad et al. 2014). Moreover, foreign body responses are likely triggered, a process thought to be driven by macrophages not able to phagocytose the crystals, leading to granuloma formation as an attempt to encapsulate the particles, which might be the scenario in the present case.

Histological studies commonly distinguish three regions along the root canal when reporting findings: the coronal, middle and apical thirds (Minic et al. 2022; Peng et al. 2017). The coronal third typically represents the earliest stage of mineralisation, characterised by collagen fibre matrix deposition, with biomaterial-induced calcifications often observed in close proximity to the materials used. The middle third is characterised by more advanced mineralisation, with calcified islands that may fuse to form a thin layer of cementum. The apical third exhibits the most advanced stage of mineralisation, with the formation of a thick acellular matrix resembling cementum. These histological patterns often correspond to radiographic findings demonstrating increases in root length, dentinal wall thickness and apical closure (Minic et al. 2022). In the present case, intracanal mineralised tissue was observed along the dentinal walls and around the scaffold.

These findings are consistent with previous immunohistological studies following RET, which reported that newly formed tissues within the root canal showed very limited similarity to native tissues (Torabinejad et al. 2014; Digka et al. 2020). These studies suggested that repair, or a combination of repair and regeneration, rather than true regeneration, had taken place, which agrees with the present findings. In this case, the newly formed mineralised tissues consisted primarily of bone- and dentin-like tissue along the root walls, predominantly in the apical third of the pulp space. Ingrowth of bony tissue from the external root surface into the internal root canal space in the apical third was observed. Furthermore, ingrowth of resorptive tissue into the pulp space, resulting in replacement resorption, was identified.

Despite the widespread application of RET in the management of immature necrotic teeth and the high survival rates reported in numerous studies (Shaik et al. 2021), the predictability of histological outcomes remains unclear. Current scientific evidence regarding histological outcomes after RET in humans indicates that newly formed intracanal tissues typically consist of a mixture of cementum-like tissue and poorly mineralised bone. Significant increases in root length and root width following RET have been reported (Conde et al. 2017). The newly formed tissues consisted of cementum-like and poorly mineralised bone, showing positivity for bone sialoprotein (BSP) and negativity for dentin sialoprotein (DSP). However, these histological findings were derived exclusively from cases with clinically successful RET outcomes. Consequently, a significant knowledge gap persists regarding the histological characteristics of tissues formed in unsuccessful RET cases. Importantly, persistent bacterial infection was identified as the main cause of treatment failure. The scientific evidence of antibacterial protocols in RET finds considerable methodological heterogeneity, with wide variation in irrigation protocols, sodium hypochlorite concentrations (0.5–6%) and intracanal medicaments such as triple antibiotic paste or calcium hydroxide, limiting definitive conclusions (Digka et al. 2020; Riaz and Shah 2021).

In this case, blood clot was used as scaffold. However, there is a recognised knowledge gap regarding the influence of different scaffolds on the outcomes of RET in humans. When platelet concentrates were used as scaffolds, poorly organised tissues with no evidence of dentine- or pulp-like formation and no clear advantage over induced blood clot alone were observed (Riaz and Shah 2021). Overall, newly formed tissues differed structurally and functionally from native pulp-dentine, highlighting the limited regenerative potential of current human RET protocols.

Current evidence on scaffold influence in RET is largely derived from heterogeneous animal studies. Across systematic and scoping reviews, a consistent pattern emerges: scaffold-based RET generally supports periapical healing and some root maturation but fails to achieve predictable regeneration of a functional pulp-dentine complex (Altaii et al. 2017; Elnawam et al. 2024).

Histologically, canal tissues are predominantly reparative, with cementum- or bone-like deposition and fibrous connective tissue with vascular ingrowth, whilst true dentine formation and organised pulp-like architecture are rare (Altaii et al. 2017). Blood clot-based approaches often favour periodontal-type healing, highlighting limited odontogenic specificity of existing protocols. Scaffold composition modulates cellular responses but does not overcome these limitations: collagen-based scaffolds support hard tissue deposition, gelatine-based and cell-augmented approaches modestly improve pulp-like outcomes, and platelet concentrates primarily enhance apex maturation without organised tissue regeneration (Matoug-Elwerfelli et al. 2025). Decellularised extracellular matrix (ECM)-derived scaffolds show the most promising organisation, likely reflecting preserved biochemical cues, though inflammatory reactions. Across scaffold types, repair predominates over true regeneration, and methodological heterogeneity and risk of bias limit comparability and clinical translation (Elnawam et al. 2024).

### Presence of inflammatory cells in the newly formed tissues after RET

The role of inflammation as a contributing factor in tissue repair and regeneration has been described previously (Matoug-Elwerfelli et al. 2025). Inflammatory infiltration of newly formed soft tissues with polymorphonuclear antigen-presenting leukocytes has been reported in traumatic injury, regardless of persistent bacterial infection after RET (Digka et al. 2020). Other studies have demonstrated that unsuccessful RET cases in children with traumatic dental injuries may exhibit persistent inflammatory cells even in the absence of detectable bacterial infection (Elnawam et al. 2024).

In the present case, an inflammatory reaction within the pulp space was also observed. Moderate levels of inflammation have been shown to stimulate new tissue formation (Altaii et al. 2017), and may represent a defence mechanism aimed at preventing the spread of residual infection from the root canal to the periapical tissues (Cooke 2019). However, because the functional role of the inflammatory infiltrate remains poorly understood, it is currently unclear whether such an immune response contributes to the long-term immunocompetence of the newly formed tissues.

### The impact of luxation injuries on the RET outcome

The root resorption observed in this case, with ingrowth of resorptive tissue into the pulp space resulting in replacement resorption, is most likely a sequela of the initial luxation injury to the periodontal ligament (Andreasen et al. 1995). Ankylosis-related replacement root resorption has been reported as the most severe complication following lateral luxation injuries in growing individuals (Tsilingaridis et al. 2012; Meschi et al. 2019). This condition is progressive and leads to infra-positioning of the affected tooth, accompanied by arrested growth of the surrounding alveolar bone. Therefore, the progression of ankylosis-related replacement root resorption may itself have had a decisive influence on the long-term prognosis of the tooth and should be considered the primary cause of the unsuccessful RET outcome.

Treatment of traumatic dental injuries during early adolescence, a period characterised by extensive transitional changes and growth of the dentoalveolar apparatus, presents a significant clinical challenge. Ongoing facial growth, eruption of immature teeth, and development of the alveolar processes complicate treatment planning, necessitating individualised clinical judgement and a multidisciplinary approach. In the present case, treatment options such as decoronation and autotransplantation were evaluated to manage the infraocclusion of tooth 11. However, uncertainty regarding the nature of the tissues within and surrounding the RET-treated tooth favoured extraction followed by interim prosthetic rehabilitation.

The clinical relevance of these findings underscores that when cellular processes cannot be adequately controlled, there is a risk of unfavourable outcomes. This consideration is particularly important in cases involving severe luxation injuries and should be carefully integrated into treatment planning.

## Conclusion

Regenerative endodontic treatment in this traumatised immature permanent incisor resulted in the formation of heterogeneous intracanal tissues with limited similarity to native odontogenic tissues. Inflammatory root resorption and ankylosis-related replacement resorption were observed, likely related to the initial luxation injury. The identification of cholesterol crystal deposits represents a novel histological finding and may provide new insight into post-RET tissue dynamics. Given the limited control over cellular events, RET outcomes remain unpredictable, particularly in cases involving periodontal ligament trauma.

## Data Availability

No datasets were generated or analysed during the current study.

## References

[CR1] Abbott PV. Prevention and management of external inflammatory resorption following trauma to teeth. Aust Dent J. 2016;61:82–94. 26923450 10.1111/adj.12400

[CR2] Abela GS, Nidorf SM (eds). Cholesterol crystals in atherosclerosis and other related diseases. Contemporary Cardiology. Humana, Cham; 2023

[CR3] Altaii M, Richards L, Rossi-Fedele G. Histological assessment of regenerative endodontic treatment in animal studies with different scaffolds: a systematic review. Dent Traumatol. 2017;33:235–44. 10.1111/edt.12338. 28342218 10.1111/edt.12338

[CR4] Andreasen JO, Borum MK, Jacobsen HL, Andreasen FM. Replantation of 400 avulsed permanent incisors. 4. Factors related to periodontal ligament healing. Endod Dent Traumatol. 1995;11:76–89. 7641622 10.1111/j.1600-9657.1995.tb00464.x

[CR5] Byers MR, Närhi MV. Dental injury models: experimental tools for understanding neuroinflammatory interactions and polymodal nociceptor functions. Crit Rev Oral Biol Med. 1999;10(1):4–39. 10.1177/10454411990100010101. 10759425 10.1177/10454411990100010101

[CR6] Chen MY, Chen KL, Chen CA, Tayebaty F, Rosenberg PA, Lin LM. Responses of immature permanent teeth with infected necrotic pulp tissue and apical periodontitis/abscess to revascularization procedures. Int Endod J. 2012;45(3):294–305. 22077958 10.1111/j.1365-2591.2011.01978.x

[CR7] Conde MCM, Chisini LA, Sarkis-Onofre R, Schuch HS, Nör JE, Demarco FF. A scoping review of root canal revascularization: relevant aspects for clinical success and tissue formation. Int Endod J. 2017;50:860–74. 10.1111/iej.12711. 27770435 10.1111/iej.12711

[CR8] Cooke JP. Inflammation and its role in regeneration and repair. Circ Res. 2019;124:1166–8. 10.1161/CIRCRESAHA.118.314669. 30973815 10.1161/CIRCRESAHA.118.314669 PMC6578588

[CR9] Digka A, Sakka D, Lyroudia K. Histological assessment of human regenerative endodontic procedures (REP) of immature permanent teeth with necrotic pulp/apical periodontitis: a systematic review. Aust Endod J. 2020;46:140–53. 10.1111/aej.12371. 31432612 10.1111/aej.12371

[CR10] Elnawam H, Abdallah A, Nouh S, Khalil NM, Elbackly R. Influence of extracellular matrix scaffolds on histological outcomes of regenerative endodontics in experimental animal models: a systematic review. BMC Oral Health. 2024;24:511. 10.1186/s12903-024-04266-x. 38689279 10.1186/s12903-024-04266-x PMC11061952

[CR11] Iwaya SI, Ikawa M, Kubota M. Revascularization of an immature permanent tooth with apical periodontitis and sinus tract. Dent Traumatol. 2001;17(4):185–7. 11585146 10.1034/j.1600-9657.2001.017004185.x

[CR12] Kim S, Suvarna CL, Bancroft JD. Bancroft’s theory and practice of histological techniques. 8th ed. Elsevier - Churchill Livingstone; 2018.

[CR13] Lin LM, Rosenberg PA. Repair and regeneration in endodontics. Int Endod J. 2011;44(10):889–906. 21718337 10.1111/j.1365-2591.2011.01915.x

[CR14] Lin JC, Lu JX, Zeng Q, Zhao W, Li WQ, Ling JQ. Comparison of mineral trioxide aggregate and calcium hydroxide for apexification of immature permanent teeth: a systematic review and meta-analysis. J Formos Med Assoc. 2016;115(7):523–30. 26911724 10.1016/j.jfma.2016.01.010

[CR15] Matoug-Elwerfelli M, Alghutaimel H, Hasan J, Nazzal H. Scaffold-based regenerative endodontics: a scoping review of orthotopic regeneration outcomes. Adv Paed Dent. 2025;1:31–53.

[CR16] Meschi N, Hilkens P, Van Gorp G, Strijbos O, Mavridou A, de Canas Llano Perula M, et al. Regenerative endodontic procedures posttrauma: immunohistologic analysis of a retrospective series of failed cases. J Endod. 2019;45:427–34. 10.1016/j.joen.2019.01.007. 30833096 10.1016/j.joen.2019.01.007

[CR17] Minic S, Vital S, Chaussain C, Boukpessi T, Mangione F. Tissue characteristics in endodontic regeneration: a systematic review. Int J Mol Sci. 2022;23:10534. 10.3390/ijms231810534. 36142446 10.3390/ijms231810534 PMC9504778

[CR18] Möller AJ, Fabricius L, Dahlén G, Ohman AE, Heyden G. Influence on periapical tissues of indigenous oral bacteria and necrotic pulp tissue in monkeys. Scand J Dent Res. 1981;89:475–84. 6951246 10.1111/j.1600-0722.1981.tb01711.x

[CR19] Murray PE, Garcia-Godoy F, Hargreaves KM. Regenerative endodontics: a review of current status and a call for action. J Endod. 2007;33(4):377–90. 17368324 10.1016/j.joen.2006.09.013

[CR20] Nagata JY, Gomes BP, Rocha Lima TF, Murakami LS, de Faria DE, Campos GR, et al. Traumatized immature teeth treated with 2 protocols of pulp revascularization. J Endod. 2014;40(5):606–12. 24767551 10.1016/j.joen.2014.01.032

[CR21] Nagendrababu V, Chong B, McCabe P, Shah P, Priya E, Jayaraman J, et al. PRICE 2020 guidelines for reporting case reports in endodontics: a consensus-based development. Int Endod J. 2020;53:619–26. 10.1111/iej.1328. 32090342 10.1111/iej.13285

[CR22] Nair PN. Cholesterol as an aetiological agent in endodontic failures—a review. Aust Endod J. 1999;25:19–26. 10.1111/j.1747-4477.1999.tb00063.x. 11411072 10.1111/j.1747-4477.1999.tb00063.x

[CR23] Nair PN, Sjogren U, Sundqvist G. Cholesterol crystals as an etiological factor in non-resolving chronic inflammation: an experimental study in Guinea pigs. Eur J Oral Sci. 1998;106:644–50. 9584911 10.1046/j.0909-8836.1998.eos106206.x

[CR24] Nanci A. Ten Cate’s oral histology: development, structure, and function. 10th ed. Elsevier - Health Sciences Division; 2024.

[CR25] Nosrat A, Li KL, Vir K, Hicks LM, Fouad A. Is pulp regeneration necessary for root maturation? J Endod. 2013;39:1291–5. 24041394 10.1016/j.joen.2013.06.019

[CR26] Paukner K, Králová Lesná I, Poledne R. Cholesterol in the cell membrane—an emerging player in atherogenesis. Int J Mol Sci. 2022;23:533. 35008955 10.3390/ijms23010533 PMC8745363

[CR27] Peng C, Zhao Y, Wang W, Yang Y, Qin M, Ge L. Histologic findings of a human immature revascularized/regenerated tooth with symptomatic irreversible pulpitis. J Endod. 2017;43:905–9. 10.1016/j.joen.2017.01.031. 28416306 10.1016/j.joen.2017.01.031

[CR28] Pereira T, Naik S, Tamgadge A. Quantitative evaluation of macrophage expression using CD68 in oral submucous fibrosis: an immunohistochemical study. Ann Med Health Sci Res. 2015;5(6):435–41. 10.4103/2141-9248.177983. 27057383 10.4103/2141-9248.177983 PMC4804656

[CR29] Petti S, Glendor U, Andersson L. World traumatic dental injury prevalence and incidence, a meta-analysis—one billion living people have had traumatic dental injuries. Dent Traumatol. 2018;34:71–86. 29455471 10.1111/edt.12389

[CR30] Riaz AA, Shah F. Regenerating the pulp-dentine complex using autologous platelet concentrates: a critical appraisal of the current histological evidence. Tissue Eng Regen Med. 2021;18:37–48. 10.1007/s13770-020-00291-3. 33150561 10.1007/s13770-020-00291-3 PMC7862478

[CR31] Robertson A, Andreasen FM, Andreasen JO, Norén JG. Long-term prognosis of crown-fractured permanent incisors. The effect of stage of root development and associated luxation injury. Int J Paediatr Dent. 2000;10(3):191–9. 10.1046/j.1365-263x.2000.00191.x. 11310111 10.1046/j.1365-263x.2000.00191.x

[CR32] Samstad EO, Niyonzima N, Nymo S, Aune MH, Ryan L, Bakke SS, et al. Cholesterol crystals induce complement-dependent inflammasome activation and cytokine release. J Immunol. 2014;192(6):2837–45. 10.4049/jimmunol.1302484. 24554772 10.4049/jimmunol.1302484 PMC3985066

[CR33] Schmalz G, Widbiller M, Galler KM. Clinical perspectives of pulp regeneration. J Endod. 2020;46:161–74. 10.1016/j.joen.2020.06.037. 10.1016/j.joen.2020.06.037 32950188

[CR34] Shaik I, Tulli M, Unnam P, Karunakaran S, Vaddi DS, Jabeen R, et al. Regenerative endodontic therapy in the management of nonvital immature permanent teeth: a systematic review and meta-analysis. J Pharm Bioallied Sci. 2021;13:36–42. 10.4103/jpbs.JPBS_807_20. 10.4103/jpbs.JPBS_807_20 PMC8375786 34447039

[CR35] Thibodeau B, Teixeira F, Yamauchi M, Caplan DJ, Trope M. Pulp revascularization of immature dog teeth with apical periodontitis. J Endod. 2007;33(6):680–9. 17509406 10.1016/j.joen.2007.03.001

[CR36] Torabinejad M, Faras H, Corr R, Wright KR, Shabahang S. Histologic examinations of teeth treated with 2 scaffolds: a pilot animal investigation. J Endod. 2014;40(4):515–20. 24666902 10.1016/j.joen.2013.12.025

[CR37] Tsilingaridis G, Malmgren B, Andreasen JO, Malmgren O. Intrusive luxation of 60 permanent incisors: a retrospective study of treatment and outcome. Dent Traumatol. 2012;28(6):416–22. 10.1111/j.1600-9657.2011.01088.x. 22107160 10.1111/j.1600-9657.2011.01088.x

[CR38] Wikström A, Brundin M, Romani Vestman N, Rakhimova O, Tsilingaridis G. Endodontic pulp revitalization in traumatized necrotic immature permanent incisors: early failures and long-term outcomes—a longitudinal cohort study. Int Endod J. 2022;55(6):630–45. 35332566 10.1111/iej.13735 PMC9325385

